# COVID-19 Outbreak on a Passenger Ship and Assessment of Response Measures, Greece, 2020

**DOI:** 10.3201/eid2707.210398

**Published:** 2021-07

**Authors:** Sophia Hatzianastasiou, Varvara A. Mouchtouri, Androula Pavli, Maria Tseroni, Spyros Sapounas, Charalampos Vasileiou, Katerina Dadouli, Maria Kyritsi, Michalis Koureas, Panagiotis Prezerakos, Matthaios Speletas, Georgios Panagiotakopoulos, Sotirios Tsiodras, Christos Hadjichristodoulou

**Affiliations:** Hellenic National Public Health Organization, Athens, Greece (S. Hatzianastasiou, A. Pavli, M. Tseroni, S. Sapounas, G. Panagiotakopoulos);; University of Thessaly Faculty of Medicine, Larisa, Greece (V.A. Mouchtouri, K. Dadouli, M. Kyritsi, M. Koureas, M. Speletas, C. Hadjichristodoulou);; European Union Healthy Gateways Joint Action, Larisa (V.A. Mouchtouri, C. Hadjichristodoulou);; Department of Hygiene Inspections of the Port of Piraeus, Region of Attica, Athens (C. Vasileiou);; University of Peloponnese Department of Nursing, Tripoli, Greece (P. Prezerakos);; University of Patras Faculty of Medicine, Patra, Greece (G. Panagiotakopoulos);; National and Kapodistrian University Medical School, Athens (S. Tsiodras)

**Keywords:** COVID-19, coronavirus disease, SARS-CoV-2, severe acute respiratory syndrome coronavirus 2, viruses, respiratory infections, zoonoses, antibodies, cruise, ship, ferry, quarantine, isolation, outbreak, asymptomatic, investigation, mask

## Abstract

We describe response measures to an outbreak involving 128 (33.4%) coronavirus disease cases (46.1% asymptomatic) among 383 persons onboard a passenger ship. Multivariate analysis indicated that dining in certain rooms and bar areas, nationality, working department (for crew members), and quarantining onboard the ship were significantly associated with infection.

On March 7, 2020, a passenger ship (2,500-passenger and 1,606-bed capacity) with 33 crew members sailed from Piraeus, Greece, to Cesme, Turkey, where an additional 350 crew members embarked on March 8, 2020 (*1*). For 21 days, the ship sailed without any disembarkations or embarkations until the first suspected coronavirus disease (COVID-19) case was reported to the health authority of the Piraeus port on March 28, 2020. We describe results of the outbreak investigation, including risk factors for transmission of severe acute respiratory syndrome coronavirus 2 (SARS-CoV-2).

## The Study

We collected data by completing standardized forms through interviews and medical examination of all travelers onboard and by reviewing the ship records and logs. We used descriptive statistics to analyze the study variables and performed univariate and multivariate analyses.

In conducting clinical management of cases, we followed guidelines from the Hellenic National Public Health Organization (NPHO) for health measures on travelers and repatriation, which were based on the European Union Healthy Gateways Joint Action advice for management of COVID-19 cases onboard ships (*2*) (Appendix Table 1). NPHO and the Piraeus Port Health Authority provided passengers with information about using medical facemasks at all times when outside of their cabins, as well as handwashing, physical distancing, and cleaning and disinfecting of cabins; ship officers supervised.

Food preparation and laundry and cleaning services were halted; travelers were instructed to clean their own cabins and store used linens in plastic bags. Cleaning and disinfection of the terminal was done by a private company under supervision of the Piraeus Port Health Authority, after all travelers disembarked the ship at the port of Piraeus. A catering company provided packaged meals; personal hygiene supplies were also provided (including facemasks and hand sanitizer). Methods and results of the environmental sampling have been published elsewhere (*3*).

We collected oropharyngeal specimens from all travelers onboard. Molecular tests for SARS-CoV-2 detection were performed by using the Cobas SARS-CoV-2 test qualitative assay and the Cobas 6800/8800 System (La Roche, https://www.roche.com). Serologic tests were performed on blood specimens collected from 116 cases. Serum samples were initially tested with the Xiamen Boson Biotech (https://www.bosonbio.com) Rapid 2019-nCoV IgG/IgM Combo Test Card, a rapid lateral flow (immunochromatographic) test, and subsequently with the MAGLUMI800 chemiluminescence immunoassay (Snibe Diagnostic, https://www.snibe.com).

Our study was a public health response as part of activities of the Hellenic NPHO and local authorities (i.e., Piraeus Port Health Authority and Port Administration). Participants provided verbal informed consent for recording and processing of information during interviews, and written consent was obtained from participants for blood specimen analysis. All required ethics considerations were applied according to rules of the Hellenic NPHO and the Ministry of Health.

The first 3 symptomatic cases occurred on March 20 among travelers (passengers and crew) of different nationalities and working departments (hotel, dining room service, and housekeeping [cabin steward]). The peak of the outbreak occurred during March 30–April 1 (Figure). We conducted laboratory tests for SARS-CoV-2 and for antibodies during 3 follow-up examinations (Appendix Table 2).

**Figure:**
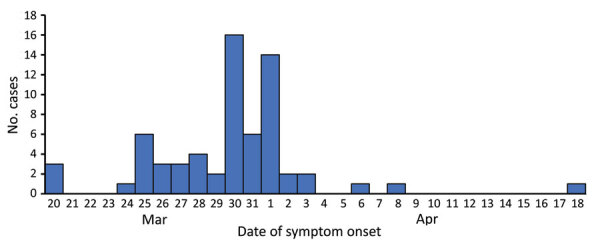
Epidemic curve for symptomatic coronavirus disease case-patients, by date of symptom onset, during an outbreak on a passenger ship, Greece, March 20–April 18, 2020. A total of 65 case-patients had a known date of symptom onset.

Travelers who tested positive were isolated onboard (except the first case-patients, who were hospitalized, and 2 travelers who were isolated in hotels designated by the government of Greece for that purpose). All travelers onboard who tested negative were considered contacts and quarantined individually in quarantine facilities ashore (hotels designated by the government of Greece), except 36 crew members who tested negative but quarantined in separate decks and facilities onboard to ensure safe ship operation. We compiled characteristics of travelers, hospitalizations, and quarantine measures (Appendix Table 2), symptom frequency (Appendix Table 3), and results of univariate analysis for testing risk factors (Appendix Table 4). No deaths occurred; 7 patients were hospitalized, including the first patient, who was intubated. 

We conducted multivariate analysis, in the form of binary logistic regression, using SPSS 25.0 (IBM, https://www.ibm.com). For all analyses, we used a 5% significance level. Multivariate analysis results indicated that test-negative travelers quarantined at hotels had lower odds of SARS-CoV-2 infection than those who quarantined onboard the ship (odds ratio 0.07, 95% CI 0.01–0.58). Travelers of nationality A who worked in the entertainment department had higher odds of infection (odds ratio 3.54, 95% CI 1.03–12.16). Multivariate analysis indicated that dining in certain rooms and bar areas, nationality, working department (for crew members), and quarantine onboard the ship significantly associated with infection (Table).

**Table:** Multivariate analysis of risk factors for SARS-CoV-2 infection during an outbreak on a passenger ship, Greece, March 20–April 18, 2020*

Factor	Area of work (for crew members)			Consuming meals and drinks in galley/dining area/bar A‡	
	Food and beverage	Housekeeping and hotel	Other†		
Nationality					
A	Referent				
B	0.00 (0.00–0.00)	NA	NA		NA
C	1.20 (0.51–2.81)	**0.11 (0.02–0.75)**	**0.03 (0.002–0.42)**		**0.49 (0.24–0.99)**
D	0.81 (0.36–1.83)	0.23 (0.05–1.14)	NA		0.54 (0.27–1.08)
E	0.90 (0.25–3.21)	0.96 (0.10–9.27)	NA		0.94 (0.32–2.78)
Other†	1.51 (0.41–5.62)	1.02 (0.14–7.20)	0.22 (0.02–2.98)		1.52 (0.59–3.91)
Consuming meals and drinks in galley/dining area/bar A‡					
	Referent	**2.71 (1.34–5.46)**	**4.44 (1.81–10.95)**	NA	

*A total of 120 passengers and crew tested positive during the first specimen collection and testing. Values in bold type are statistically significant. SARS-CoV-2, severe acute respiratory syndrome coronavirus 2; NA, not applicable. †Entertainment, security, shop, hospital, other. ‡Meals and beverages were prepared and served in galley and dining room and café-bar A (for travelers who embarked at Cesme port) and in galley and dining room and café-bar B (for travelers who embarked at Piraeus port).

## Conclusions

Our findings can be used in COVID-19 prevention and control preparedness plans for ports and ships. Ongoing transmission can occur onboard ships, affecting a large number of travelers, without any sign of symptoms for an extended period, especially in cases where most of the travelers are of young age. Assuming a serial interval number of 5 days and a reproduction number of 2.6, we could conclude that 1 infectious traveler embarked on the first or second day of the voyage, and 21 days later 120 travelers on board had been infected (*4*,*5*).

Active or passive surveillance for COVID-19 based on symptoms onboard ships alone cannot be effective for early COVID-19 outbreak detection because only a small proportion of cases can be identified when the disease has already spread widely. Screening of incoming travelers and preboarding and regular routine laboratory testing can be implemented in addition to surveillance (*6*). We advise laboratory diagnostic testing of passengers and crew for SARS-CoV-2 within 72 hours before embarkation and a second test the day of embarkation. In addition, for crew members, we advise a 10-day quarantine period before starting work and regular testing of all crew members on board every 7 days. The preparedness plans of ships and ports should ensure laboratory capacity for diagnosis of all persons onboard once an outbreak has been identified and frequent and regular testing until negative results are obtained in accordance with port policies (*7*).

Because of the large proportion of travelers who tested positive in this outbreak, the relevant authorities decided that COVID-19 case-patients would be isolated onboard the ship, whereas travelers who tested negative would disembark and quarantine individually in hotels. Individual quarantine and isolation in separate facilities was effective in preventing further spread (*2*,*6*,*8*–*11*). These measures contributed to preventing intra-cabin transmission, which was documented in other COVID-19 outbreaks onboard passenger ships (*12*,*13*). Halting food preparation and service, housekeeping activities in cabins, and laundry service stopped transmission onboard. This outcome is contrary to the outbreak management approach taken onboard another cruise ship, where crew continued working duties while passengers and crew were isolated in their cabins, resulting in further COVID-19 spread (*11*). In our study, logistic regression statistical analysis showed that quarantining travelers in hotels was a protective factor against SARS-CoV-2 infection compared with quarantining onboard the ship.

Crew members who worked in the entertainment department, were of a certain nationality, and consumed meals and drinks at specific dining and bar areas had a higher risk for infection. Dining areas and bars can be settings for COVID-19 transmission because of congregation of persons and because facemasks are not used while eating or drinking (*13*). Avoiding self-service, encouraging service of meals in cabins, staggering meal times, and reconfiguring dining room seating to ensure physical distancing are recommended to avoid possible transmission within ships’ dining rooms (*2*,*11*,*14*).

A coordinated approach from a country’s central government, in cooperation with local port authorities, is needed to define the maximum response capacities of each port and the maximum number of ships that can be allowed to call. This approach will help to avoid confining travelers on ships for quarantine and isolation and reduce the risk for spread onboard when outbreaks occur, in line with the World Health Organization’s International Health Regulations, which are designed to prevent unnecessary interference with international traffic and trade (*7*).

Appendix Additional information about COVID-19 outbreak on a passenger ship and assessment of response measures, Greece, 2020.
