# Supplementary material for: COVID-19 Outbreak on a Passenger Ship and Assessment of Response Measures, Greece, 2020
Source: Emerg Infect Dis. 2021 Jul;27(7):1927–30. doi: 10.3201/eid2707.210398 (PMC8237900; doi:10.3201/eid2707.210398)
Supplement: Appendix — Additional information about COVID-19 outbreak on a passenger ship and assessment of response measures, Greece, 2020. [file 21-0398-Techapp-s1.pdf]

Article DOI: https://doi.org/10.3201/eid2707.210398

COVID-19 Outbreak on a Passenger Ship and Assessment of Response Measures, Greece, 2020

Appendix

Appendix Table 1. Risk categorization of COVID-19 patients according to travelers' characteristics*

Risk category†	Characteristics (demographics, signs and symptoms)
Low risk	Asymptomatic or Mild symptoms (fever <38.50 C and/or cough and/or sore throat) and No underlying conditions and Age <65 y and Respiratory rate ≤16/min or SaO2 >94%
Moderate risk	Symptoms: fever <38.50°C and/or cough and/or sore throat and Underlying condition or Age >65 y or Abnormal chest x-ray and Respiratory rate ≤16/min or SaO2 >94%
High risk	Symptoms: fever ≥38.5°C and/or cough and/or tiredness and/or dyspnea and Underlying condition or Age over >65 y or Abnormal chest x-ray

*The ship medical doctor performed daily health monitoring of all persons on board, while four visits for medical examinations and specimen collection from all persons on board were conducted by the public health authority's team. A second medical and nursing team performed health monitoring of travelers quarantined in hotels.
†125 /128 patients were categorized into the low risk group, while two and one were categorized into the moderate and high risk group, respectively.

Appendix Table 2. Characteristics of symptomatic and asymptomatic travelers on board the passenger ship and laboratory results

Characteristics	Frequency (%)
Age, Male/Female, Total	
20–30	156 (52.9), 44 (50.6), 200 (52.4)
31–40	67 (22.7), 25 (28.7), 92 (24.1)
41–50	40 (13.6), 14 (16.1), 54 (14.1)
51–60	27 (9.2), 3 (3.4), 30 (7.9)
61–70	4 (1.4), 1 (1.1), 5 (1.3)
71–80	1 (0.3), 0 (0.0), 1 (0.3)
Total	295 (100.0), 87 (100.0), 382 (100.0)
Mean age (min-max, SD)	33.2 (19–73, 11.5), 31.8 (20–64, 9.8), 32.9 (19–73, 11.1)
Sex	
Female	87 (22.7)
Male	296 (77.3)
Total	383 (100)
Nationality	
A	152 (39.7)
B	36 (9.4)
C	73 (19.1)
D	82 (21.4)
E	17 (4.4)
Other	23 (6.0)
Total	383 (100.0)
Embarkation port	
Piraeus	33 (8.6)
Cesme	350 (91.4)
Total	383 (100.0)
Working department	
Food and beverage	211 (55.1)
Housekeeping and Hotel	103 (26.9)
Deck and Engine	31 (8.1)
Other*	38 (9.9)
Total	383 (100.0)
SARS-CoV-2 RT-PCR test results	
Positive	128 (33.4)
Negative	255 (66.6)
Total	383 (100.0)
Positive SARS-CoV-2 RT-PCR test results	
1st examination 30–31/03/2020	120 (93.7)

Characteristics	Frequency (%)
2nd examination 14/04/2020	45† (35.1)
3rd examination 21–22/04/2020	16 (12.5)
4th examination 04/05/2020	1 (0.8)
Total	128 (100.0)
IgG antibodies test results according to the number of days passed since the onset of symptoms	
Asymptomatic	19/55
Symptomatic <5 d	1/1
Symptomatic 6–10 d	4/21
Symptomatic 11–15 d	17/30
Symptomatic >15 d	5/9
Total	46/116 (39.6)
IgM antibodies test results according to the number of days passed since the onset of symptoms‡	
Asymptomatic	11/55
Symptomatic <5 d	1/1
Symptomatic 6–10 d	1/21
Symptomatic 11–15 d	5/30
Symptomatic >15 d	4/9
Total	22/116 (19.0)
Symptom status	
Symptomatic	69 (53.9)
Asymptomatic	59 (46.1)
Total	128 (100.0)
Hospitalizations	7 (5.5)
Deaths	0 (0.0)
Place of quarantine for contacts	
Ship	36 (13.7)
Hotel	227 (86.3)
Total	263 (100.0)
Place of isolation for cases	
Ship	117 (98.3)
Hotel	2 (1.7)
Total	119 (100.0)

*Entertainment (N = 23), security (N = 8), shop (N = 4), supernumerary (N = 2), hospital (N = 1)
†Including 8 new cases
‡Clinical specimens were collected on April 10 2020.

Appendix Table 3. Symptoms frequency (N = 69)

Symptoms	Frequency	% Proportion (95%CI)
Low-grade fever (<38.5°C)	22	31.9 (21.2–44.2)
Fever (≥38.5°C)	15	21.7 (12.7–33.3)
Cough	23	33.3 (22.4–45.7)
Sore throat	12	17.4 (9.3–28.4)
Headache	12	17.4 (9.3–28.4)
Muscle aches and pains	10	14.5 (7.2–25.0)
Dyspnea with strenuous exercise	0	0 (0.0–5.2)
Dyspnea with no exercise	1	1.5 (0.0–7.8)
Diarrhea	5	7.3 (2.4–16.1)
Abdominal pain	3	4.4 (0.9–12.2)
Vomiting	0	0 (0.0–5.2)
Anosmia	14	20.3 (11.6–31.7)
Ageusia	1	1.5 (0.0–7.8)
Other	9	13.0 (6.1–23.3)

Appendix Table 4. Testing for risk factors of becoming infected using univariate analysis* (N = 120 who tested positive during the first specimen collection)

Factors	Relative Risk (95%CI)	p value
Gender (Male/ Female)	1.18 (0.81–1.72)	0.392
Work position		
Bar service	Reference	
Dining room service	**2.33 (1.16–4.69)**	**0.007**
Cook	1.45 (0.70–3.01)	0.299
Cabin steward	0.54 (0.19–1.52)	0.234
Utility	0.50 (0.14–1.72)	0.310
Department of work		
Housekeeping and Hotel	Reference	
Food and beverage	**2.41 (1.49–3.91)**	**<0.001**
Deck and Engine	1.04 (0.41–2.61)	0.999
Other†	**3.39 (1.97–5.83)**	**<0.001**
Nationality		
B	Reference	
A	**3.08 (1.34–7.09)**	**0.001**
C	1.58 (0.63–3.97)	0.317
D	1.49 (0.60–3.73)	0.380
E	2.54 (0.90–7.17)	0.073
Other	**3.44 (1.37–8.63)**	**0.004**
Nationality and work department		
Food and beverage & Nationality A	Reference	
Entertainment & Nationality A	**1.78 (1.16–2.73)**	**0.042**
Food and beverage & Nationality D	0.81 (0.47–1.39)	0.552
Housekeeping & Nationality D	**0.29 (012–0.67)**	**0.001**
Food and beverage & Nationality C	1.00 (0.61–1.64)	0.999
Food and beverage & Nationality E	0.86 (0.37–1.97)	0.767
Port of embarkation (Cesme/ Piraeus)	2.17 (0.95–4.93)	0.018
Galley and dining place A/ Galley and dining place B	**2.39 (1.04–5.46)**	**0.018**

*Means and standard deviations (SD) were reported for continuous variables, while frequencies and proportions with 95% Confidence Intervals (CI) were reported for categorical variables. Proportions' 95% CI were calculated based on binomial distribution. Data were also checked for deviation from normal distribution using the Shapiro-Wilk normality test. Categorical data were analyzed with the use of Chi-square tests and Fisher exact tests as appropriate; Student's t-tests and Mann-Whitney U tests were used for continuous data as appropriate. Univariate analysis was conducted by calculating the relative risk (RR) of becoming infected with SARS-CoV-2 with the following factors: gender, age, work position, department of work, nationality, port of embarkation, dining area, place of quarantine, and deck number.
†Entertainment, security, shop, supernumerary, hospital.
